# Burden of type 2 diabetes attributed to lower educational levels in Sweden

**DOI:** 10.1186/1478-7954-9-60

**Published:** 2011-12-16

**Authors:** Emilie E Agardh, Anna Sidorchuk, Johan Hallqvist, Rickard Ljung, Stefan Peterson, Tahereh Moradi, Peter Allebeck

**Affiliations:** 1 Department of Public Health Sciences, Division of Social Medicine, Karolinska Institutet, Stockholm, Sweden; 2 Department of Public Health and Caring Sciences, Uppsala University, Uppsala, Sweden; 3 Department of Public Health Sciences, Division of Public Health Epidemiology, Karolinska Institutet, Stockholm, Sweden; 4 Department of Molecular Medicine and Surgery, Karolinska Institutet, Stockholm, Sweden; 5 Department of Public Health Sciences, Division of International Health Care Research, Karolinska Institutet, Stockholm, Sweden; 6 Department of Environmental Medicine, Division of Epidemiology, Karolinska Institutet, Stockholm, Sweden; 7 St Petersburg State Medical Academy named after II. Mechnicov, Division of Epidemiology, St Petersburg, Russia

## Abstract

**Background:**

Type 2 diabetes is associated with low socioeconomic position (SEP) in high-income countries. Despite the important role of SEP in the development of many diseases, no socioeconomic indicator was included in the Comparative Risk Assessment (CRA) module of the Global Burden of Disease study. We therefore aimed to illustrate an example by estimating the burden of type 2 diabetes in Sweden attributed to lower educational levels as a measure of SEP using the methods applied in the CRA.

**Methods:**

To include lower educational levels as a risk factor for type 2 diabetes, we pooled relevant international data from a recent systematic review to measure the association between type 2 diabetes incidence and lower educational levels. We also collected data on the distribution of educational levels in the Swedish population using comparable criteria for educational levels as identified in the international literature. Population attributable fractions (PAF) were estimated and applied to the burden of diabetes estimates from the Swedish burden of disease database for men and women in the separate age groups (30-44, 45-59, 60-69, 70-79, and 80+ years).

**Results:**

The PAF estimates showed that 17.2% of the diabetes burden in men and 20.1% of the burden in women were attributed to lower educational levels in Sweden when combining all age groups. The burden was, however, most pronounced in the older age groups (70-79 and 80+), where lower educational levels contributed to 22.5% to 24.5% of the diabetes burden in men and 27.8% to 32.6% in women.

**Conclusions:**

There is a considerable burden of type 2 diabetes attributed to lower educational levels in Sweden, and socioeconomic indicators should be considered to be incorporated in the CRA.

## Background

A considerable number of studies show that low socioeconomic position (SEP) is associated with disease, and socioeconomic inequalities in morbidity and mortality are highlighted as key concerns in many countries [1-4]. The World Health Organization (WHO) Comparative Risk Assessment (CRA) module of the Global Burden of Disease (GBD) study is widely used as a unified systematic approach to assess the contribution of selected risk factors to disease burden all over the world [5,6]. CRA includes 26 risk factors, mainly based on behavioral and medical risk factors such as physical inactivity, body mass index (BMI), smoking, blood pressure, and cholesterol. The included risk factors are selected on the basis of the following criteria: likely to be among the leading global or regional causes of disease burden; not too specific or too broad; have high likelihood of causality; availability of reasonably complete data on exposure and risk levels; and potentially modifiable [5], i.e., all in line with Bradford Hills' criteria [7].

No socioeconomic indicators are included in the CRA. For example, in the case of the effect of education on health, the argument is that the effect is dependent on other socioeconomic factors and on the policy context, which includes accessibility and effectiveness of health and welfare systems, and therefore the theoretical minimum risk exposure distribution is likely to change over time and space [8]. In addition, the causality between socioeconomic indicators and disease can be questioned, and whether they should be regarded as risk factors, mediators, or confounders can be debated [9]. However, socioeconomic inequalities in health have been acknowledged in the scientific literature for more than 30 years [3,10], and a socioeconomic gradient for many diseases such as coronary heart disease and type 2 diabetes also persists after control of confounders [9,11]. The etiological role of SEP in heart disease has been conceptualized as SEP producing both direct effects (or through direct pathways of chronic stress) [9,12], as well as indirect effects mediated by traditional risk factors for disease [9]. By this view, socioeconomic factors should be treated as the "causes of the causes" of heart disease. Although the theoretical minimum risk exposure may change over time and space, and SEP is a distal factor in the causal web of risk factors, the important role of SEP in the development of many diseases cannot be neglected [3]. Including socioeconomic indicators into the well-known CRA and GBD framework will not only assess and map their contribution to disease burden but also visualize their role in public health debates. In this study, we therefore attempt, as a first step, to illustrate an example by estimating the burden of type 2 diabetes in Sweden attributed to lower educational levels as a measure of SEP using the methods applied in CRA. By this example and by the discussion of our findings and limitations, we hope that further efforts and debates will emerge about the possibilities of including socioeconomic indicators in CRA and burden of disease estimates.

## Methods

### Principles for Comparative Risk Assessment (CRA)

The contribution of risk factors to disease burden in CRA are based on meta-level estimates of risk factor and disease relationships from the international epidemiological literature combined with estimates of the risk factor distribution in the population of interest [5,8]. The risk factor and disease relationship is assumed to be similar across different populations in the world, while the distribution of exposure differs between societies. Thus, the risk of developing type 2 diabetes due to physical inactivity or obesity is the same for everyone, but the prevalence of physical inactivity or obesity differs between societies. For each risk factor, CRA expert groups have re-analyzed data from published and unpublished primary data, original data, systematic reviews, and meta-analyses to obtain unified estimates between different risk factors and diseases.

The estimates of disease burden due to risk factors are based on a counterfactual exposure distribution in which the lowest exposure level is the theoretical minimal risk exposure, irrespective if currently attainable in practice [5]

### The association between lower educational levels and type 2 diabetes incidence

We used data from our recent systematic review and meta-analysis to obtain estimates of the relative risk (RR) of educational levels and type 2 diabetes incidence [11]. Briefly, in that study, we systematically searched relevant case-control and cohort studies published in English between January 1966 to January 2010 in PubMed and EMBASE using the key words diabetes and educational level, occupation, or income. Out of 5120 citations, 23 studies (41 measures of associations) were included in a meta-analysis after fulfilling the following inclusion criteria: i) presented original data using case-control or cohort study design; ii) provided information about type 2 diabetes as an outcome, iii) presented risk estimates with confidence intervals (CIs) or sufficient information to calculate these; iv) used educational level, occupation, or income as individual measures of SEP. The included studies were from countries all over the world, and the levels of SEP included in the studies varied from two to five categories. Some studies reported separate analyses for men and women, and some combined both sexes.

For the purpose of this study, we restricted the previously mentioned inclusion criteria further and only included data from the articles that met the following criteria: i) included at least three levels of exposure; ii) included information on men and women separately; and iii) were performed in high-income countries, according to the World Bank definition. Due to limited number of studies providing the above information using occupation and income as measures of SEP, we only included educational level in this study. Moreover, we restricted our analysis to high-income countries, due to lack of data from low- and middle-income countries. Five studies (six measures of association) fulfilled this restricted inclusion criteria [13-17] (Table 1).

**Table 1:** Studies included in the meta-analysis; for more detailed information, see original meta-analysis [11]

First author, year	Setting	Study period	Ages*	Sex	RR (for low and middle versus high educational level) (95% CI)	Variables controlled for
Kouvonen A et al., 2008 [14]	Cohort study, Finland	1986-2004	18-65	Men	High (≥13 yrs): 1.00 Middle (10-12 yrs): 0.91 (0.63-1.30) Low (≤9 yrs): 1.39 (0.98-1.97)	Age by adjustment
Medalie J et al., 1974 [16]	Cohort study, Tel Aviv, Israel	1963-1968	40+	Men	High (≥13 yrs): 1.00 Middle (10-12 yrs): 1.35 (1.00-1.83) Low (≤9 yrs): 1.63 (1.22-2.18)	None^a^
Robbins J et al., 2005 [17]	Cohort study (NHANES), US	1980-1992	29-84	Men Women	High (≥13 yrs): 1.00 Middle (10-12 yrs): 1.19 (0.86-1.63) Low (≤9 yrs): 1.52 (1.15-2.01) High (≥13 yrs): 1.00 Middle (10-12 yrs): 1.37 (1.02-1.83) Low (≤9 yrs): 2.24 (1.71-2.94)	None^a^ None^a^
Kaye S et al., 1991 [13]	Nested case-control study, Iowa, US	1985-1987	55-69	Women	High (≥13 yrs): 1.00 Middle (10-12 yrs): 1.25 (0.97-1.61) Low (≤9 yrs): 2.00 (1.51-2.65)	Age by adjustment
Lidfeldt J et al., 2007 [15]	Cohort study (the Nurse's Health Study), US	1992-2002	46-71	Women	High (≥15 yrs): 1.00 Middle (13-14 yrs): 1.34 (1.19-1.52) Low (≤12 yrs): 1.79 (1.61-2.0) High (≥15 yrs): 1.00 Middle (13-14 yrs): 1.14 (1.01-1.29) Low (≤12 yrs): 1.16 (1.04-1.29)	Age by adjustment Age, BMI, physical activity, quintile of dietary score, alcohol consumption, smoking baseline hypertension + hypercholesterolemia, FHD, menopausal, use of HRT, ethnicity, birth weight, breastfeeding, and childhood SEP by adjustment

^a ^Risk estimate calculated from crude data

*Ages at baseline or at diagnosis, for cases only or total, depending on reporting, given as mean or age range

RR = relative risk, CI = confidence interval, BMI = body mass index, WHR = waist-hip-ratio, FHD = family history of diabetes, HRT = hormone replacement therapy, SEP = socioeconomic position

Educational level was classified into high (≥13 years), middle (10 to 12 years) and low (≤9 years), using high educational level as the reference group. In all studies except one [15] the reference group was ≥13 years of schooling. The exception used ≥15 years as the reference group. To minimize the influence of potential confounders we used the most adjusted estimates (Table 1). However, some studies only reported crude estimates, and some reported only age-adjusted. To obtain one summary measure for lower educational levels and type 2 diabetes incidence RRs from individual studies were pooled using random-effects models quantifying the associations. Statistical heterogeneity among studies was evaluated using both the *Q *statistic and the *I^2^*statistics [18] (Table 2).

**Table 2:** Pooled estimates for lower educational levels and incidence of type 2 diabetes in high-income countries

Pooled RRs (95% CIs); *p-*value for the heterogeneity *Q *test, and *I^2 ^*statistics (%)			
	High (≥13 yrs)*	**Middle (10-12 yrs)**	**Low (≤9 yrs)**
***Men ***	1.00	1.16 (0.93-1.44); *p *= 0.26, *I^2 ^*= 26.4	1.52 (1.28-1.82); *p *= 0.79, *I^2 ^*= 0.0
***Women***	1.00	1.18 (1.07-1.31); *p *= 0.47, *I^2 ^*= 0.0	1.71 (1.07-2.74); *p *= 0.001, *I^2 ^*= 93

*One study used ≥15 years as the reference group [15].

### Prevalence of educational levels in Sweden

For information on population distribution of educational levels in Sweden we collected prevalence data from the Swedish population in 2008 using the longitudinal integration database for health insurance and labor market studies (LISA) [19]. To ensure comparability, the same classification of education was used for Swedish data as we found in the systematic reviews and meta-analyses from the international literature. Thus, educational level was treated as a categorical variable with three categorizations, i.e., high (≥13 years), middle (10-12 years) and low (≤9 years). According to the CRA procedure, we first stratified data into the following age groups (0-4, 5-15, 16-29, 30-44, 45-59, 60-69, 70-79, and 80+ years) for men and women separately. We excluded ages 0 to 15 years, since no data on educational levels were available for these age groups, and also 16 to 29 years. The reason for this is that those between the ages 16 and 19 automatically will be categorized into lower educational groups due to their ages, and they have therefore no opportunity to obtain higher levels of education. This in combination with the low incidence of type 2 diabetes in the age group 15 to 19 could lead to misleading results (Table 3).

**Table 3:** Estimated prevalence (%) of educational levels in Sweden by sex and age groups

Educational levels	16-29*	30-44	45-59	60-69	70-79	80+	Total
***Men ***							
High (≥13 yrs)	19.4	37.2	30.6	25.6	18.7	14.1	27
Middle (10-12 yrs)	43.7	49.5	48.4	41.8	34.8	30.6	45
Low (≤9 yrs)	32.3	11.1	20.0	31.6	45.0	53.0	26
No data	4.7	2.2	0.9	1.0	1.5	2.4	2
***Women***							
High (≥13 yrs)	27.0	47.1	37.2	28.3	17.6	8.8	32
Middle (10-12 yrs)	39.0	42.6	47.9	43.7	35.2	26.1	41
Low (≤9 yrs)	29.9	8.7	14.1	27.0	45.3	61.5	25
No data	4.1	1.6	0.8	1.1	1.9	3.6	2

Data on educational levels are collected from the Swedish population in 2008, using the longitudinal integration database for health insurance and labor market studies (LISA) [19].

*This age group was excluded from further calculations.

### Burden of diabetes mellitus

We used the data on diabetes burden from our latest available GBD study in 2002. The data, which is inserted in a database (WHO toolkit), comprise estimates from the global burden of diabetes mellitus in the year 2000 [20]. Representative population-based studies using oral glucose tolerance tests and 1980 WHO criteria to define diabetes prevalence by age and sex were used. Since the majority of studies did not indicate the type of diabetes, the estimates refer to all diabetes. To derive internally consistent estimates of age-specific incidence patterns for diabetes, published prevalence and incidence studies were applied within Dismod II (a software tool developed by WHO to check the consistency of estimates of incidence, prevalence, duration, and case fatality for diseases). Sweden is part of region EURO A, and the data were based on published studies from this region, although they were adjusted to Swedish population estimates from United Nation Population Division from 2003 [21]. Diabetes mortality estimates were based on the Swedish National Cause of Death Register from 2003. The burden of disease in the GBD study is presented by disability-adjusted life years (DALYs), a measure that quantifies the gap between a population's current health and an ideal situation in which everyone lives to old age in full health. DALYs combine two single indicators, years of life lost to disability (YLD) and years of life lost from premature death (YLL). YLD is calculated by multiplying the incidence of a disease (I) by expected time for recovering or death (L) and a disability weight (DW) that has a value from 0 (full health) to 1 (death), depending on diagnosis, YLD = I × L × DW. YLL is calculated by multiplying numbers of death due to a specific disease (N) with expected life expectancy at time of death (L), YLL = N × L. The burden of disease estimates (YLDs, YLLs, and DALYs) that we present in this study are neither age-weighted nor discounted. Hence, time lived is valued the same for all ages, and lives saved at present have not been valued higher then lives saved in the future. The disability weights included in YLD were based on the GBD Study 1990 [21].

### Data analysis: population attributable fraction (PAF)

To assess type 2 diabetes attributed to lower educational levels, we calculated population attributable fractions (PAFs) in Excel spreadsheets using the pooled RRs of the association between type 2 diabetes incidence and lower educational levels from our meta-analysis, as well as the prevalence of educational levels in Sweden from LISA. PAFs were calculated using the following formula [8]:

$$PAF=\frac{\sum_{i=1}^{n}P_{i}\left(RR_{i}-1\right)}{\sum_{i=1}^{n}P_{i}\left(RR_{i}-1\right)+1}$$

The prevalence of exposure (*P_i_*) was assessed for high, middle, and low education, and each was multiplied with the pooled RR (*RR_i_*) of type 2 diabetes in its own corresponding exposure level (high, middle, and low) minus 1, which represents the excess risk attributed to the exposure levels. Confidence intervals for PAFs were obtained using nonparametric bootstrapping [22]. For each of the five parameters in the PAF (high, middle, and low prevalence of education and middle and low RRs), 10,000 estimates were iteratively sampled from each parameter's respective distribution.

PAF is an expression of the percentage reduction in population disease or death that would occur if exposure to a risk factor was reduced to an ideal exposure scenario [8]. This approach yields estimates of potential gains in population health that would occur if exposure to risk factors was reduced from all suboptimal levels. As in CRA, we assumed that the counterfactual exposure distribution was based on the principle of the theoretical minimal risk, which in our study was high educational level, and we thus investigated how much of the type 2 diabetes burden in Sweden would be reduced if the whole population had high educational level. The PAFs were then applied to the Swedish burden of diabetes estimates of death, YLLs, YLDs, and DALYs (by multiplying the PAFs with diabetes deaths, YLLs, YLDs, and DALYs) [8] overall and within the predefined age groups as described above, excluding the age group 15 to 29 years, for reasons described previously.

## Results

### Type 2 diabetes incidence and low educational level

In men, the pooled RR for middle and low compared to high educational level was 1.16 (95% CI: 0.93-1.44) and 1.52 (1.28-1.82), and in women the corresponding estimates were 1.18 (1.07-1.31) and 1.71 (1.07-2.74) (Table 2).

In men, a moderate heterogeneity was observed for middle educational level, although no heterogeneity was observed for low educational level. In women, no heterogeneity was observed in the estimates of middle educational level, while there was a high heterogeneity for low educational level (Table 2).

### Prevalence of educational levels in Sweden

As presented in Table 3, 26% of men in Sweden have a low educational level, 45% middle, and 27% high. The corresponding prevalences for women were 25%, 41%, and 32%.

### The burden of type 2 diabetes attributable to low educational levels

In men, 17.2% (7.9%-26.2%) of the burden of diabetes was attributed to lower educational levels, and in women 20.1% (7.6%-33.8%) of the burden could be explained by lower educational levels (Table 4). It was apparent in both men and women that the burden of diabetes attributable to lower educational levels was most pronounced in older age groups (60-80+), which is a result of population distribution of educational levels. When we applied the PAF estimates to the diabetes burden, 3,099 out of 18,055 DALYs in men (data not shown in table) were attributed to lower educational levels, as were 3,499 out of 17,424 DALYs in women (data not shown in table).

**Table 4:** The burden of diabetes attributable to lower educational levels by sex and age groups in Sweden

	Men					Women				
	**Deaths**	**YLLs**	**YLDs**	**DALYs**	**PAF (%), CIs**	**Deaths**	**YLLs**	**YLDs**	**DALYs**	**PAF (%), CIs**

**Total** **(all ages)**	163	1671	1428	3099	17.2%, 7.9-26.2	209	1796	1755	3499	20.1%, 7.6%-33.8%
**30-44**	2	72	153	225	12.0%, 2.1%-21.8%	1	45	126	171	12.2%, 6.0%-19.5%
**45-59**	14	389	562	951	15.4%, 5.7%-24.9%	6	170	467	637	15.7%, 7.4%-25.5%
**60-69**	18	311	349	660	18.8%, 9.7%-27.6%	16	329	437	766	21.3%, 8.1%-35.5%
**70-79**	58	582	238	820	22.5%, 13.1%-31.2%	58	695	454	1149	27.8%, 8.4%-45.5%
**80+**	120	576	115	691	24.5%, 15.0%-33.3%	231	1066	312	1378	32.6%, 8.1%-52.8%

YLLs = years of life lost, YLDs = years lived with disability, DALYs = disability-adjusted life years, PAFs = population attributable fractions (expressed as percentages)

Data on educational levels are collected from the Swedish population in 2008 using the longitudinal integration database for health insurance and labor market studies (LISA)[19]. Data on diabetes are collected from GBD Study 2002 [20].

## Discussion

We have illustrated an example in which we estimated the burden of type 2 diabetes attributed to lower educational levels using the methods applied in CRA. From our results, it was apparent that lower educational levels accounted for 17% of the Swedish diabetes burden in men and 20% of the burden in women. Although there are limitations of including socioeconomic indicators in CRA, these results indicate that a considerable burden of type 2 diabetes can be attributed to lower educational levels in Sweden. There are reasons to believe that the same would apply for many other diseases or for other socioeconomic indicators, such as occupation or income.

This is, to our knowledge, the first published attempt to assess the contribution of a socioeconomic indicator using the CRA methodology, although previous studies have used PAFs as a measure of explaining social inequalities in health [23,24]. Much work is devoted to estimating burden of disease and to assessing which risk factors can be reduced in order to prevent morbidity and mortality [21]. Type 2 diabetes is an example of a disorder that itself is a risk factor for several disorders, and is thus a part of a causal chain between distal risk factors, such as low educational level and income, proximal risk factors, such as obesity and physical inactivity, physiological risk factors, such as high cholesterol, hypertension, and type 2 diabetes, and endpoints, such as ischemic heart disease [8,25]. By this view, many entry points for intervention are possible. Including socioeconomic factors into the well-known CRA and GBD framework may highlight the effect of distal risk factors and give incentives to interventions at more distal levels in the causal web of risk factors.

Although it may be questioned if SEP should be regarded as a risk factor for type 2 diabetes, many studies have discussed and confirmed an association between type 2 diabetes and low SEP [11,26]. For the purpose of this study, we performed a comprehensive systematic review of more than 5,000 citations to investigate and summarize the published research about SEP and incidence of type 2 diabetes. The strength of associations was consistent in high-income countries. However, it became clear that we only could estimate this association in high-income countries, due to lack of data from middle- and low-income countries [11]. From the perspective of the epidemiological transition in middle- and low-income countries, where economic development leads to the emergence of noncommunicable disease among the more affluent groups [27], it is likely that a reverse scenario would appear. Although this issue needs further investigation and clarification with regard to type 2 diabetes, it seems as if different estimates will be needed for different economies.

We only used educational level as a measure of SEP because of reasons described previously. Indicators such as educational level may have different meanings and classifications for different birth cohorts and geographical settings, even if limited to certain regions or economies. This could be problematic when combining international data. On the other hand, educational level has the advantage of being relatively easy to measure and is relevant to people regardless of working circumstances [28]. Also, education is usually obtained early in life, i.e., prior to onset of type 2 diabetes, which can prevent drawing misleading conclusions on reverse causality. In addition, the classification of educational levels is rather similar in high-income countries, as the lowest level most likely corresponds to nine-year compulsory school, not to illiteracy. In the meta-analysis that we used for this study, it was clear that the categorizations of educational level from the international literature corresponded well to each other as well as to the Swedish setting, regardless of the time of collection of data. Unfortunately, we could not perform a more refined educational classification on the prevalence data for Sweden, since we had to ensure comparability with the data from the meta-analysis.

Another important issue is that only five studies, including six measures of association [13-17], met the inclusion criteria in the meta-analysis that we used in this study, compared to our original meta-analysis with 23 risk estimates (from 20 studies). Hence, the representativeness of the estimates to high-income countries from these few articles can be questioned. On the other hand, the pooled estimates we used in this study corresponded rather well to the estimates from the original meta-analysis, even if that meta-analysis included more data from various high-income countries. For example, in the original meta-analysis, the increased risk of type 2 diabetes for women with lowest versus highest educational level was 72% (RR = 1.72, 95% CI: 1.26-2.35) compared to 71% (1.71, 1.07-2.74) in this study. For men, the results differed slightly from the original study with an increased risk of 46% (1.46, 1.15-1.86) in the original meta-analysis compared to 52% (1.52, 1.28-1.82) in the present study.

It should be further discussed if combining data from different geographical settings is accurate. Espelt et al. have reported that although lower educational levels are associated with diabetes prevalence and mortality all over Europe, there are differences between countries [29]. For example, Eastern European countries have higher relative inequalities in mortality than the rest of Europe. On the other hand, in our original meta-analysis we observed a moderate heterogeneity in the overall association between type 2 diabetes incidence and SEP when pooling data from various high-income countries, which indicates the possibility of combining SEP data. One option to overcome difficulties in combining data from different geographical settings could be to estimate burden of disease using country-specific estimates of the association of SEP and disease incidence. For example, one previous study based on Swedish prevalence data showed that about one-third of the measured disease burden was attributed to adverse SEP in men [30]. However, one advantage of CRA is that it enables mapping and assessing risk factors to disease burden in a unified way [5]. Furthermore, in our original meta-analysis, one study from Sweden was included [31], although it only compared low versus high educational level. In this Swedish study, the increased RR for low compared to high educational level was 70% in women and 40% in men. Hence, even in this case the estimates were rather similar to the pooled estimates from both the original meta-analysis, as well as from the restricted meta-analysis that we used in this study. To assess the stability of the results both in our original meta-analysis and when pooling RRs for the present study, we performed leave-one-out influence analysis [32]. In this method, the potential influence of one individual study on the overall pooled RR was assessed by omitting one study at a time. The results showed that no individual study significantly altered the overall estimates, neither in the original [11] or present analysis.

We have not performed a systematic review and meta-analysis on type 2 diabetes and mortality and consequently have not distinguished between incidence and mortality in the YLL and YLD estimates. It is most likely that a pooled RR of mortality would differ from the incidence of type 2 diabetes. For example, the RR of dying due to diabetes in low SEP groups in Europe is twice as high in men and 3.4 times higher in women compared to those with high SEP [26,29]. These results indicate a higher RR for mortality than for incidence. If applying these RRs in the PAF estimates for YLL, the role of low educational level on diabetes burden would be even higher.

Another issue is the influence of potential confounders. Although we used the most adjusted estimate from each study, the estimates ranged from crude to multi-adjusted, and the RRs may be overestimated or underestimated to some extent, which could lead to an overestimated or underestimated contribution of lower educational levels to diabetes burden. This issue was addressed in the original meta-analysis by subanalyzing multi-adjusted and minimally-adjusted estimates separately and with a sensitivity analysis. Those results showed that the increased RRs persisted for multi-adjusted estimates even if the effect was decreased. It is also possible that the risk factors adjusted for are intermediates in the causal pathway between educational levels and type 2 diabetes incidence. However, a possible adjustment for intermediates would lead to overadjustment and to an underestimated pooled RR and consequently to underestimated contribution of lower educational levels to diabetes burden.

It should also be noted that we have not estimated the association between type 2 diabetes incidence and educational levels for different age groups. It is possible that the risk is lower or higher at younger ages, but also that the risk is diluted or strengthened in the elderly. A previous study reported that educational inequalities in mortality decreased gradually with age in men (30-90+); however, in women the association was rather stable from ages 40-49 to 70-79 and then decreased from 80-89 to 90+ [33]. Thus, if the same pattern applied for type 2 diabetes incidence, the PAFs may be overestimated to some extent, at least in the older age groups.

The country-specific data that we used for prevalence of educational levels were collected from LISA 2008, a unique representative population-based register covering all individuals from 16 years of age registered in Sweden [19]. Educational levels have in general been increasing during the past decades in Sweden, and if this increase continues, less burden of diabetes may be attributed to lower educational levels. However, we do not know much about future educational inequalities and projecting future associated burden is beyond the scope of this study.

For the YLL, YLD, and DALY estimates we have relied on data from the WHO toolkit. Diabetes mortality was based on the Swedish National Cause of Death Register and thus the YLLs represent the best available data on diabetes mortality in Sweden. However, for incidence, prevalence, and duration of diabetes mellitus, data from Sweden are derived from published studies from region EURO A. Thus, our burden of diabetes estimates, especially YLDs but also DALYs, are constrained by the limitations of the GBD study.

Finally, diabetes in the GBD study refers to all diabetes [20]. Although it has been estimated that type 2 diabetes accounts for 90% to 95% of all cases [34], some type 1 diabetes cases that have a different pattern with regard to SEP are likely to be included in the estimations. However, since we did not include people in the age range 0 to 29 years, in which most cases are type 1 diabetes we do not believe this would influence the results substantially.

## Conclusion

There is a considerable burden of type 2 diabetes attributed to lower educational levels in Sweden. Although we believe that the results from the present study give a good indication of the attributable role of lower educational levels to burden of type 2 diabetes in Sweden, the PAFs should be interpreted cautiously. More studies are needed on the association between type 2 diabetes incidence and educational levels, as well as occupation and income, in order to decide whether they should be included as risk factors for type 2 diabetes in the GBD study. With more studies, it will also be easier to discuss if a combined estimate from different countries is appropriate to use, or whether socioeconomic indicators should be based on risk estimates from separate countries or regions. We also suggest that before omitting socioeconomic indicators from the CRA framework, further and more refined studies based on systematic reviews of socioeconomic indicators in association with other diseases should be performed.

## Competing interests

The authors declare that they have no competing interests.

## Authors' contributions

TM, PA, and JH came up with the conception of including SEP as a risk factor in CRA. TM prepared the WHO toolkit for statistical analysis and implanted the practical knowledge for further use in the group. EA, PA, JH, TM, and AS carried out the systematic review and meta-analysis. EA and AS performed the statistical analyses. EA drafted the manuscript, and all authors (EA, AS, JH, RL, SP, TM, and PA) interpreted and discussed the results and were involved in critically revising the manuscript. All authors (EA, AS, JH, RL, SP, TM, and PA) read and approved the final manuscript.
